# Perinatal outcomes of pregestational hypertension according to blood pressure range at 11–14 week scan: Impact of the 2017 ACC/AHA guidelines

**DOI:** 10.3389/fmed.2022.994386

**Published:** 2022-10-14

**Authors:** Alvaro Sepúlveda-Martínez, Tomas Conrads, Rodolfo Guiñez, Javiera Guiñez, Marcelo Llancaqueo, Mauro Parra-Cordero

**Affiliations:** ^1^Maternal and Fetal Medicine Unit, Department of Obstetrics and Gynecology, Hospital Clínico de la Universidad de Chile, Santiago, Chile; ^2^Maternal and Fetal Medicine Unit, Department of Obstetrics and Gynecology, Hospital Clínico San Borja Arriarán, Santiago, Chile; ^3^Maternal and Fetal Medicine Unit, Department of Obstetrics and Gynecology, Universidad del Desarrollo y Clínica Alemana de Santiago, Santiago, Chile; ^4^Critical Care Unit, Department of Medicine, Hospital Clínico de la Universidad de Chile, Santiago, Chile; ^5^Cardiology Unit, Department of Internal Medicine, Hospital Clínico de la Universidad de Chile, Santiago, Chile

**Keywords:** hypertension, pregnancy, perinatal outcomes, blood pressure, ACC/AHA guidelines

## Abstract

**Objective:**

The aim of this study was to evaluate the impact on perinatal outcomes related to placental insufficiency with the application of the new 2017 ACC/AHA guidelines to a group of chronic hypertensive pregnancies during their first-trimester assessment.

**Study design:**

This retrospective cohort study included pregnancies with preconceptional hypertension and known perinatal outcomes. In the first trimester, a combined screening for preterm preeclampsia (p-PE) was performed, including blood pressure (BP), mean uterine artery Doppler, and maternal characteristics. Patients were divided, according to the 2017 ACC/AHA consensus, into the following groups: elevated or less, Stage 1, and Stage 2. For adverse perinatal outcome assessment, univariate and multivariate regression analyses were performed, considering the “elevated or less” group as a reference. Odds ratios (OR) were compared with linear trend analysis. The main outcomes measured were preterm PE and FGR < 3*^rd^* percentile.

**Results:**

Of the 130 included patients, 59 (45.4%) were classified as elevated or less, 47 (36.2%) as Stage 1, and 24 (18.4%) as Stage 2. p-PE showed a significant increase according to BP range [7% (OR = 1.0), 19.6% (OR = 3.2), and 21.7% (OR = 3.7)]; trend p = 0.02, for elevated or less, Stage 1, and Stage 2, respectively. There was a non-significant increased trend of FGR < 3*^rd^* percentile according to the BP stage. The best multivariate predictive model for p-PE included a previous PE background (OR = 15) and mean arterial pressure in mmHg (OR = 1.1).

**Conclusion:**

The use of the 2017 ACC/AHA consensus in pregnancies with chronic hypertension identifies an intermediate risk group for placental-mediated diseases.

## Introduction

Preeclampsia (PE) affects 5% of pregnancies worldwide (1), accounting for 10–15% of direct maternal deaths (2, 3). Severe PE is associated with the presence of seizures, acute pulmonary edema, stroke, acute kidney injury, liver failure, and disseminated intravascular coagulopathy (4). As for the fetus, PE can lead to fetal growth restriction (FGR) and indicate preterm birth (5). One of the most significant risk factors for PE is chronic hypertension (crHT), increasing the risk around five times (6). crHT is observed in around 1–5% of all pregnancies (7, 8). Pregnant women with crHT also have increased pregnancy-related morbidity for other pathologies, such as preterm delivery, stroke, FGR, placental abruption, and stillbirth (7–10).

In 2017, the American Heart Association (AHA) and the American College of Cardiology (ACC) published practice guidelines that changed the definition of stage 1 hypertension, lowering the systolic blood pressure (SBP) and diastolic blood pressure (DBP) values (11). The new definition was made after the evaluation of several meta-analyses that showed that crHT and its cardiovascular complications were already significant with SBP of 130–139 and DBP of 85–89 mmHg vs. <120/80 mmHg. Thus, the definition is intended to promptly counsel and intervene in cardiovascular health (12).

Nonetheless, the implications of this newly diagnosed stage 1 hypertension were unclear for the obstetric practice. There are only few studies regarding whether pregnant women with stage 1 hypertension had a higher risk of maternal and perinatal complications. In 2018, a cohort study was published, which observed higher rates of complications for women with the new stage 1 hypertension (13). However, more studies are needed to establish a stronger association. Additionally, the American College of Obstetricians and Gynecologists (ACOG) has not changed its guidelines regarding pharmacological antihypertensive treatment recommendations (14).

Therefore, the objective of this study was to evaluate the impact on perinatal outcomes associated with placental insufficiency, by the application of the new 2017 ACC/AHA guidelines to a group of chronic hypertensive pregnant women during their assessment of the first trimester of pregnancy.

## Materials and methods

### Study design

This retrospective cohort study was conducted at the Fetal Medicine Unit Hospital Clínico Universidad de Chile from November 2011 to June 2020. Before the 11–14 week screening scan, all patients were asked to complete a demographic questionnaire that included chronic disease background (pregestational hypertension, diabetes mellitus, autoimmune diseases, thrombophilia, chronic renal disease, and thyroid disease), family history of PE, and pregnancy background (parity, previous PE, and previous fetal growth restriction). When a chronic disease was reported, treatment was asked for and stored in the dedicated database Astraia^®^.

Later, the current weight (kilograms) and height (centimeters) were obtained. Blood pressure was measured according to the Seventh Report of the Joint National Committee (JNC-7) using a validated digital sphygmomanometer, Omron HEM-7113 (Omron Healthcare Inc. Illinois, USA). Blood pressure was measured simultaneously in both arms and stored. For preterm PE (p-PE) screening, the multivariable algorithm of O′Gorman *et al.* (15) was used, with the software Astraia^®^ (Astraia software gmbh, Ismaning, Germany). Briefly, maternal history was combined with mean uterine artery pulsatility index (UtA-PI) and mean arterial pressure (MAP), both expressed as a multiple of the median (MoM) (16, 17). A high risk of p-PE was considered with a combined risk greater than 1/100. In these cases, low-dose aspirin was recommended (18).

Perinatal outcomes were obtained from a local electronic medical chart or delivery registries. Aspirin use during pregnancy, and gestational age at the onset of PE was obtained from the electronic medical chart. In the case of delivery in another institution, patients were contacted *via* phone to obtain perinatal data. First-trimester screening assessment and perinatal data were stored in the software Astraia^®^ and exported to an excel sheet for depuration before analysis.

### Patient′s selection

For this study, all singleton pregnancies with known pregestational chronic hypertension during the study period were included. All patients used oral antihypertensives before conception. Withdrawal or treatment modification was performed at the first pregnancy control by an OB&GYN or maternal-fetal medicine specialist. Newborns with major birth defects or aneuploidies were excluded from the analysis. Patients without a blood pressure measurement at the first-trimester screening ultrasound and/or without a known perinatal outcome were also excluded.

### Definitions

For this study, the following definitions according to the ACC/AHA guidelines (11) were used to classify our cohort prospectively: Normal blood pressure (BP) is defined as systolic blood pressure (SBP) of <120 mmHg and diastolic blood pressure (DBP) of <80 mmHg; elevated BP as SBP between 120 and 129 mmHg and DBP < 80 mmHg; stage 1 hypertension was considered as an SBP between 130 and 139 mmHg and/or diastolic blood pressure (DBP) between 80 and 89 mmHg. Stage 2 was considered with an SBP of 140 mmHg or more and/or a DBP of 90 mmHg or more.

Mean arterial pressure was estimated using the following formula: MAP = DBP + [(SBP-DBP)/3], where DBP was the mean diastolic blood pressure of both arms and SBP was the mean systolic blood pressure of both arms.

Preeclampsia was diagnosed following the 2019 NICE consensus (19), and p-PE was defined as an antenatal PE with delivery before 37 weeks of gestation. Global preterm delivery (PTD) was defined as delivery by any cause before 37 weeks and global early PTD as delivery before 34 weeks. Fetal growth restriction (FGR) was diagnosed with a birthweight < 3*^rd^* centile (20), according to fetal curves proposed by Hadlock (21). Small for gestational age was considered as a birthweight of < 10*^th^* centile. Placental-mediated diseases were considered as the presence of PE and/or FGR < 3*^rd^* percentile.

### Statistical analysis

For all statistical analyses and figure designs, the dedicated software Stata^®^ version 16.1 (StataCorp, College Station, USA) was used. Continuous variables were explored using the Shapiro-Wilk test to determine variable distribution. Parametric and non-parametric variables were compared using ANOVA or Kruskal-Wallis tests and expressed as mean ± standard deviation or median (interquartile range), respectively. *Post hoc* comparisons between pairs of groups were performed using Bonferroni or Mann-Whitney tests as appropriate. Categorical variables were compared with chi (2) or Fisher exact tests and expressed as percentages.

For this study, pregnancies classified as normal or elevated blood pressure were merged into the “elevated or less” group. To determine the linear incremental risk of all perinatal outcomes, a trend analysis using the Jonckheere-Terpstra test was performed. Odds ratios were estimated using univariate logistic regression analyses, considering the elevated or less group as the reference. Finally, a multivariate logistic backward stepwise regression analysis was performed to identify whether the first-trimester blood pressure was associated with the development of severe placental-mediated diseases. For multivariate regression analysis, a p-value of 0.1 was considered for exclusion and 0.05 for inclusion in the final model. For all other statistical analyses, a p-value less than 0.05 was considered significant. STROBE guidelines were followed for study design and reporting.

## Results

**FIGURE 1:**
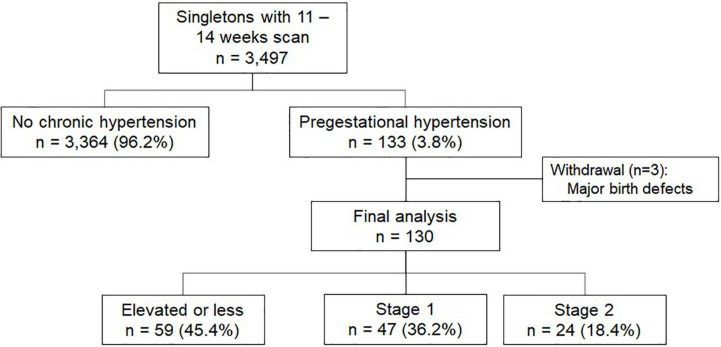
Flowchart for patient selection.

During the study period, 3,497 singleton pregnancies were assessed at 11 + 0 to 13 + 6 weeks for PE and aneuploidy screening ultrasound, using the current Fetal Medicine Foundation (FMF) algorithm, including blood pressure assessment. Of these, 133 (3.8%) were considered pregestational hypertension, based on a demographic questionnaire before the ultrasound. Three pregnancies were excluded because of a congenital birth defect (Figure 1).

### Maternal demographics

Of the 130 included pregnancies in the final analysis, maternal age at the first-trimester assessment was 35.6 (31.6–38.2) years, with a body mass index (BMI) of 30.1 (25.7–34.5) kg/m^2^. Previous PE was reported in 34/91 (37.4%) parous patients. The median gestational age at assessment was 12.7 (12.1–13.1) weeks. Notably, eighty-two (63%) patients were using the oral antihypertensive treatment and 73 (57%) patients started aspirin before 16 weeks of gestation. At first trimester assessment, blood pressure was stratified as elevated BP or less (*n* = 59; 45.4%), Stage 1 (*n* = 47; 36.2%), and Stage 2 (*n* = 24; 18.4%). Maternal demographic characteristics and first-trimester ultrasound assessment according to blood pressure range are described in Table 1.

**TABLE 1:** Demographic characteristics of pregestational hypertensive pregnancies at 11–14 week scan.

Characteristics	Elevated or less (*n* = 59)	Stage 1 (*n* = 47)	Stage 2 (*n* = 24)	*p*-value
**Demographic characteristics**				
Maternal age, *years*	37.1 (32.7 – 39.1)	33.2 (30.3 – 37.0)*	36.3 (33.2 – 39.3)^†^	0.03
Type of conception				0.67
Spontaneous	57 (96.6)	47 (100)	24 (100)	
ART	2 (3.4)	0	0	
Systemic lupus	1 (1.7)	1 (2.1)	0	1.0
APLS	1 (1.7)	0	0	1.0
Diabetes mellitus	1 (1.7)	3 (6.4)	1 (4.2)	0.56
Smoking habit	2 (3.4)	1 (2.1)	0	0.2
Nulliparous	17 (28.8)	16 (34.0)	6 (25.0)	0.06
Parous no previous PE	20 (33.9)	25 (53.2)	12 (50.0)	
Parous previous PE	22 (37.3)	6 (12.8)*	6 (25.0)	
Body mass index, *kg/m*^2^	27.7 (24.6 – 31.6)	31.2 (26.7 – 35.9)*	31.7 (29.5 – 35.2)*	0.0025
Antihypertensive therapy	35 (59.3)	31 (66.0)	16 (66.7)	0.7
Aspirin before 16 weeks	36 (61.0)	25 (53.2)	12 (54.6)	0.7
**11 – 14 weeks assessment**				
CRL, *mm*	65 (55 – 70)	64 (56 – 74)	65 (58 – 69)	0.96
Nuchal translucency, *mm*	1.6 ± 0.4	1.7 ± 0.4	1.5 ± 0.4	0.2
Mean UtA PI	1.54 (1.24 – 2.01)	1.63 (1.22 – 1.85)	1.47 (1.28 – 1.80)	0.78
UtA > 95^th^ percentile	1/59 (1.7)	3/47 (6.4)	1/23 (4.4)	0.49
MAP, *mmHg*	87 (83 – 91)	97 (95 – 99)*	107 (105 – 110)^‡^	0.0001

ART, assisted reproductive technology; APLS, antiphospholipid syndrome; p-PE, preterm preeclampsia; CRL, crown-to-rump length; UtA, uterine artery; PI, pulsatility index; MAP, mean arterial pressure; FMF, fetal medicine foundation. P-value between all groups with the Kruskal-Wallis test.

*p < 0.05 vs. elevated or less; ^†^*p* < 0.05 vs. Stage 1; ^‡^*p* < 0.05 vs. Stage 1 and normotensive.

As expected, there was a positive trend between BMI and blood pressure range, with 27.7 kg/m^2^ in the elevated or less group and 31.7 kg/m^2^ in the Stage 2 group, respectively (*p* < 0.01). There was no association between first-trimester UtA Doppler PI and blood pressure (*r* = −0.05; *p* = 0.6), with a median PI of 1.54, 1.63, and 1.47 for elevated or less, Stage 1, and Stage 2, respectively (*p* = 0.78, Table 1).

### Perinatal outcomes

**FIGURE 2:**
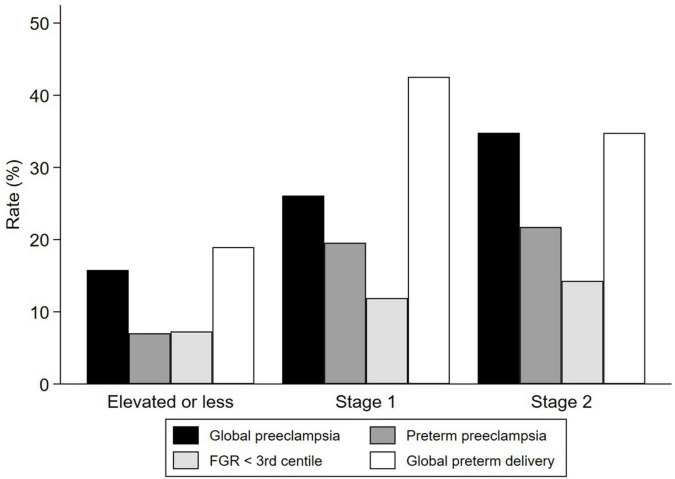
Perinatal outcomes of placental-mediated diseases according to blood pressure stage at the first-trimester scan. FGR, fetal growth restriction.

There were 3 (2.3%) fetal losses (one case in each group, *p* = 0.8). PE was observed in 29 (22.3%) patients, with a significant increasing trend regarding blood pressure range in the first trimester (Table 2). Rates of p-PE (*n* = 18; 14%) were also higher when blood pressure was higher, with an almost 3-fold and 4-fold higher risk for the Stage 1 and Stage 2 groups compared to the elevated or less group, respectively (Figure 2 and Table 2).

**TABLE 2:** Odds ratios for perinatal outcomes after univariate logistic regression analysis.

Outcome	Elevated or less (*n* = 59)		Stage 1 (*n* = 47)		Stage 2 (*n* = 24)		
	n/N (%)	OR (95% CI)	n/N (%)	OR (95% CI)	n/N (%)	OR (95% CI)	Trend *p*-value*
Global preeclampsia	9/57 (15.8)	Reference	12/46 (26.1)	1.9 (0.71 – 4.96)	8/23 (34.8)	2.8 (0.93 – 8.67)	0.03
Preterm preeclampsia	4/57 (7.0)	Reference	9/46 (19.6)	3.2 (0.92 – 11.25)	5/23 (21.7)	3.7 (0.89 – 15.22)	0.02
SGA < 10^th^ percentile	6/57 (10.5)	Reference	8/45 (17.8)	1.8 (0.58 – 5.75)	5/23 (21.7)	2.4 (0.64 – 8.69)	0.08
FGR < 3^rd^ percentile	4/55 (7.3)	Reference	5/42 (11.9)	1.7 (0.43 – 6.86)	3/21 (14.3)	2.1 (0.43 – 10.43)	0.16
Global PTD	11/58 (19.0)	Reference	20/47 (42.6)	3.2 (1.32 – 7.59)	8/23 (34.8)	2.3 (0.77 – 6.71)	0.015
Global PTD < 34wks	3/58 (5.2)	Reference	7/47 (14.9)	3.2 (0.78 – 13.17)	4/23 (17.4)	3.9 (0.79 – 18.84)	0.03

*Trend analysis with Jonckheere-Terpstra analysis. SGA, small for gestational age; FGR, fetal growth restriction; PTD, preterm delivery.

Global preterm delivery < 37 (*n* = 39; 30%) and <34 weeks (*n* = 14; 11%) were also higher in the Stage 1 and Stage 2 groups, compared to the elevated or less group (Table 2). Small for gestational age at birth showed a non-significant increase rate from 11% in the elevated or less group to 22% in the Stage 2 group (*p* = 0.08). Furthermore, the rate of FGR showed an increase related to blood pressure range (elevated or less group: 7%, Stage 1 group: 12%, and Stage 2 group: 14%), although this trend was not significant (Table 2).

Finally, a multivariable logistic regression analysis was performed in this cohort of chronic hypertensive pregnancies to identify factors related to placental-mediated diseases. Briefly, for p-PE, the independent risk factors were the history of previous PE (OR = 15) and MAP in mmHg (OR = 1.1). For FGR below the 3^rd^ percentile, the independent risk factors were UtA PI z-score (OR = 6.2), MAP in mmHg (OR = 1.1), and maternal age in years (OR = 1.4) (Table 3).

**TABLE 3:** Backward stepwise regression analysis for correlation between demographic and biophysical characteristics and presence of placental-mediated diseases.

	Up-slope Coefficient	OR (95% CI)	*p*-value	Standard error
**Preterm preeclampsia (**< 37 weeks)				
Previous preeclampsia	2.6825	14.6 (2.86 – 74.85)	0.001	12.182
MAP, *mmHg*	0.0880	1.1 (1.01 – 1.18)	0.021	0.042
Intercept	–11.3406			
**Fetal growth restriction** < 3^rd^**percentile**				
Mean UtA PI z-score	1.8229	6.2 (1.15 – 33.38)	0.034	5.322
MAP, *mmHg*	0.0910	1.1 (0.99 – 1.22)	0.08	0.053
Maternal age, *years*	0.3413	1.4 (0.94 – 2.11)	0.1	0.207
Intercept	–24.5419			

MAP, mean arterial pressure; UtA PI, uterine artery Doppler pulsatility index.

## Discussion

Our study demonstrated that the first-trimester blood pressure range in patients with pregestational hypertension is directly associated with adverse perinatal outcomes, mainly those related to placental-mediated diseases. Moreover, the new 2017 ACC/AHA classification identifies a moderate subgroup of pregnancies who could benefit from closer follow-up until delivery. Furthermore, the development of p-PE and FGR in these pregestational hypertensive pregnancies was also associated with the previous history of PE and uterine artery Doppler and maternal age, respectively.

In 2018, Nzelu et al. (22) analyzed and divided 586 pregnancies with pregestational hypertension into three groups: BP below 140/90 mmHg without treatment (Group 1), BP below 140/90 with oral antihypertensive, and BP > 140/90 irrespective of treatment at the first trimester. The goal of the treatment was to maintain BP at 130–140/80–90 mmHg, and treatment was withdrawn when BP was less than 130/80 mmHg. They demonstrated that adverse outcomes (p-PE and severe hypertension) were related to the use of antihypertensive treatments and, similar to our results, BP level in the first trimester. In this line, Webster L. *et al.* performed a longitudinal assessment of BP and vascular function parameters in 97 pregestational hypertensive pregnancies. They demonstrated that those who subsequently developed superimposed PE and SGA, SBP and DBP were significantly higher from the first trimester to delivery (23).

Similar to our results, Sutton E. et al. analyzed perinatal outcomes related to the new Stage 1 hypertension range but in low-risk pregnancies in the first trimester. They demonstrated that the lower range of Stage 1 is associated with an almost 3-fold higher risk of global PE (OR = 2.66 [1.56–4.54]) and a 4-fold higher risk of indicated preterm delivery (OR = 3.83 [1.30–11.31]) (13).

In terms of predicting p-PE and FGR, we found out that MAP was an independent predictive factor for both diseases, whereas the previous history of PE was a predictor of the former, the maternal age and uterine artery Doppler were of the latter. These results could be of interest considering the current use of multivariate predictive models, such as the FMF proposal (15). This model is applied to universal screening, where uterine artery Doppler, MAP, and maternal characteristics are the main contributors to p-PE screening. However, within the subgroup of pregestational hypertension patients, uterine artery Doppler seems to have a lower impact on p-PE prediction, compared to MAP and history of previous PE.

Despite the abovementioned evidence, several national guidelines, such as the latest ACOG Practice Guidelines, reinforce the use of the classical cutoff of 160/110 mmHg to initiate or increase antihypertensive medications in pregnancies with pregestational chronic hypertension with a treatment goal of 120–160 mmHg and 80–110 mmHg for systolic blood pressure and diastolic blood pressure, respectively (14). However, there is a lack of consensus regarding the best cutoff within this large therapeutic range. The abovementioned results of Nzelu *et* al. could help to answer this question, considering a therapeutic goal of < 140/90 mmHg as the best range to reduce adverse perinatal outcomes (22).

In contrast, the use of the new classification in non-pregnant women and early pregnancies will have a significant public health impact on rates of gestational hypertension by lowering the diagnostic cutoff. Recently, Hu J. et al. evaluated 16,345 patients in China with several blood pressure assessments across pregnancy. They demonstrated that gestational hypertension increases from 4.2 to 25.1% using the 2017 ACC/AHA consensus (24). Despite the fact that this 6-fold increase in newly diagnosed gestational hypertension could affect public health resources to manage these patients, the identification of a moderate risk population (25) could be beneficial to reduce resources due to the management of adverse perinatal outcomes.

### Strengths and weaknesses

The main strength of our study is that all patients were assessed by fetal medicine experts at 11–14 week scan with validated sphygmomanometers and all relevant data obtained before the assessment. In this line, in all included patients, the use and number of oral hypertensive treatments and the use of aspirin were obtained, with no difference between groups that could bias our results. Before statistical analyses, the complete database was depurated to certify that all data were correctly stored. However, this study is not without weaknesses. The main weakness is that our results are based on a single measurement of blood pressure, without considering a longitudinal follow-up until delivery. However, our results highlight the association between placental-mediated diseases and blood pressure range in the first trimester. Another weakness is the single institution nature of the sample, which could affect the external validation of our results. Therefore, it is important to develop a multicenter study to demonstrate consistency in our results. Moreover, because blood pressure monitoring has been mandatory in our unit just since 2016, all chronic hypertension cases before that were excluded from our analysis, reducing the sample size by almost 50%.

### Clinical relevance of the study

Our results could be of interest for the clinical management of chronic hypertension during pregnancy. By demonstrating that the new Stage 1 is associated with an increased risk of placental-mediated diseases compared to lower blood pressure values, this could support that an intensive antihypertensive strategy with a lower blood pressure threshold is beneficial not only to reduce severe FGR but also to reduce p-PE. In this line, Magee et al. demonstrated that compared to a traditional goal of diastolic blood pressure below 100 mmHg, a tight regimen (DBP below 85 mmHg) was associated with less severe hypertension during pregnancy and no increase in SGA at delivery (26). Therefore, in pregestational chronic hypertension, a treatment goal of <130/80 mmHg should be encouraged to reduce placental-mediated diseases during pregnancy. Interestingly, a recent study by Hauspurg A *et al.* (27) demonstrated that within pregnancies with a high risk of PE in the first trimester, those with the new 2017 ACC/AHA Stage 1, the use of low-dose aspirin was associated with a significant reduction in preterm PE, which was not observed in the normotensive high-risk group. These results reinforce the clinical benefit of the new classification in the management of hypertension during pregnancy that needs to be validated with a larger population of pregestational hypertensive pregnancies.

## Conclusion

Our study demonstrated that the use of the new ACC/AHA classification of chronic hypertension in the first trimester has an impact on identifying a subgroup of patients with a moderate adverse perinatal outcome that potentially requires closer follow-up and strict management during pregnancy.

## Data availability statement

The raw data supporting the conclusions of this article will be made available by the authors, without undue reservation.

## Author contributions

AS-M co-designed the study, performed the database depuration, performed all statistical analyses, and co-wrote the document. TC co-obtained perinatal outcomes and co-wrote the document. RG and JG co-obtained perinatal outcomes. ML and MP-C co-designed the study, reviewed, and approved the final draft. All authors contributed to the article and approved the submitted version.
